# Septoplasty: defining a desirable clinical outcome according to baseline symptom scores

**DOI:** 10.3389/fsurg.2025.1471526

**Published:** 2025-02-12

**Authors:** Rolf Haye, Liv Kari Døsen, Magnus TarAngen, Caryl Gay, Are Hugo Pripp, Olga Shiryaeva

**Affiliations:** ^1^ Department of Otorhinolaryngology, Lovisenberg Diaconal Hospital, Oslo, Norway; ^2^ Department of Otorhinolaryngology Head and Neck Surgery, Rikshospitalet, Oslo University Hospital, Oslo, Norway; ^3^ Department of Research, Lovisenberg Diaconal Hospital, Oslo, Norway; ^4^ Oslo Center of Biostatistics and Epidemiology, Research Support Services, Oslo University Hospital, Oslo, Norway; ^5^ Department of Patient Safety and Research, Lovisenberg Diaconal Hospital, Oslo, Norway

**Keywords:** patient outcome assessment, nasal surgical procedure, nasal obstruction, clinical audit, ROC curve

## Abstract

**Objective:**

The results of septoplasty are usually reported as statistically significant improvements in baseline scores, but these may be difficult to interpret clinically. A measure called the desirable clinically important difference (DCID) has been developed to serve as a guideline to assist in clinically interpreting improvement in scores. So far, DCID has only been calculated for whole cohorts. As individual patients have different baseline and improvement scores, such measures are not helpful to individuals. Our aim was to establish a DCID according to baseline scores, which should help assess individual results.

**Methods:**

Patients (*n* = 934) rated their nasal obstruction using a visual analog scale (VAS) preoperatively and 6 months postoperatively. A global rating of outcome (categorized as completely, much, or somewhat improved, unchanged, or worse) served as the anchor for postoperative evaluation. The improvement in VAS score corresponding to the “much improved” rating was defined as the borderline value between “much” and “somewhat improved.” Receiver operating characteristics were used to establish this borderline value. The DCID is the difference between the borderline and baseline VAS scores. The relative DCID is calculated by dividing the numeric DCID by the baseline VAS score. The cohort was divided into three subgroups (moderate, severe, very severe) according to preoperative severity of nasal obstruction (VAS score) for assessing the relation between DCID and baseline obstruction severity.

**Results:**

The DCID increased with increasing severity of baseline nasal obstruction: 27.5 (moderate), 44.5 (severe), and 56.0 (very severe), as did the relative DCID: 49.6% (moderate), 56.8% (severe), and 61.3% (very severe).

**Conclusion:**

The relative DCID can be a guide for assessing improvement following septoplasty according to baseline scores of nasal obstruction and for planning surgery. A 49% improvement from baseline is indicative of clinical success for a patient with moderately obstructed nasal breathing, whereas patients with very severe obstruction require a 61% improvement.

## Introduction

Outcomes of septoplasty are usually assessed by testing the statistical significance of symptom improvements from baseline (1–3). However, statistically significant improvements may be difficult to interpret clinically and are not necessarily clinically important (4, 5). One strategy for addressing this limitation of statistical significance is to establish the minimum improvement that is clinically important. This is called the “minimal clinically important difference” (MCID) and is often used to assess the clinical meaningfulness of septoplasty outcomes (6–8). In contrast, Ziai and Bonaparte (9) used the rating “surgical success” as clinically meaningful. They found that this rating corresponded to a 41.1% improvement in symptom scores from baseline. We have used a similar rating of “much improved” to distinguish these results from lower levels of improvement (5) and named this measure a “desirable clinically important difference” (DCID).

In septoplasty outcomes, the MCID and DCID have only been applied to whole cohorts. Patients with nasal obstruction have different baseline ratings and different grades of improvement after septoplasty. The outcomes of cohort studies are not helpful in assessing individual results. The aim of our study was therefore to assess whether the MCID and DCID differ according to baseline levels of nasal obstruction. We divided the cohort into subgroups according to baseline levels of nasal obstruction. The results from the study should help assess the improvement of obstruction ratings in individual patients and may also provide insights for preoperative planning of surgery for individual patients.

## Material and methods

Patients undergoing endonasal septoplasty, with or without turbinate surgery, are included in the Septoplasty Quality Register (SQR) at the Department of Otorhinolaryngology at Lovisenberg Diaconal Hospital, Oslo, Norway. Written informed consent is not required for inclusion of patients in the SQR in accordance with national legislation and internal requirements. We have audited anonymous data from the SQR for this study. Patients respond to the Nasal Surgical Questionnaire (NSQ) (10) preoperatively and 6 months postoperatively as part of our quality assurance policy. Between April 2014 and September 2019, 1,260 patients were entered into the register. Of these, 325 patients (26%) did not return the second questionnaire, and 1 patient omitted some ratings. The remaining 934 patients were included in this analysis. The included patients were at least 17 years of age and did not have any other nasal or sinus disease except allergy.

The questionnaire contains separate visual analog scales (VASs) for nasal obstruction during the day and at night. Each VAS consists of a 10 cm line, with the left end representing no obstruction and the right end representing complete obstruction. The patients were asked to rate their sense of nasal obstruction on each scale with a vertical line. Each VAS score is measured in mm from the left-hand side of the scale, with a range of 0–100. Patients were asked to respond to the items based on how they felt on a normal day without any infection.

The questionnaire was also mailed to each patient 6 months postoperatively, along with a cover letter signed by a surgeon from the department and a prepaid return envelope. Three weeks later, a reminder containing the same questionnaire was mailed to those who had not returned the first questionnaire. The postoperative version of the questionnaire included an additional question about the patient's retrospective sense of change in nasal obstruction following surgery: “Is your breathing now completely, much, or somewhat improved, unchanged or worse?” These global outcome ratings were assigned the following status: status 1 = completely improved, status 2 = much improved, status 3 = somewhat improved, status 4 = unchanged, and status 5 = worse.

We used the anchor-based method to establish the clinically important difference as recommended by the US Food and Drug Administration (11). Of the different statistical strategies to establish the DCID, we chose the receiver operating characteristics (ROC) approach, which establishes the borderline, maximizing specificity and sensitivity (described in the Statistical analysis section).

The minimum improvement in the VAS score corresponding to the “much improved” rating was defined as the VAS score at the borderline between status 2 (much improved) and status 3 (somewhat improved). The DCID is calculated as the difference between this borderline and the baseline VAS score.

The study cohort was large enough to be divided into three subgroups according to the patients’ baseline VAS scores of nasal obstruction: subgroup 1 (moderate obstruction) had VAS scores of 0–70, subgroup 2 (severe obstruction) had VAS scores of 71–85, and subgroup 3 (very severe obstruction) had VAS scores of 86–100.

VAS ratings of nasal obstruction are higher at night than during the day (5). As the night values better reflect the global rating of improvement, we used the night ratings for comparison with the global ratings.

### Statistical analysis

Statistical analyses were performed using IBM SPSS version 28.0 (IBM Corp, Armonk, NY, USA) and Stata statistical software (StataCorp, College Station, TX, USA). Descriptive statistics were expressed as numbers (%) and mean (SD) for patient demographics, VAS scores of nasal obstruction (both preoperative and postoperative), and outcome status groups. All of the analyses were two-sided, and statistical significance was defined as *p* < 0.05. Paired *t*-tests were used to evaluate the differences between preoperative and postoperative VAS scores for each status and nasal obstruction subgroup. *p*-values <0.05 were considered statistically significant.

The statistical plan was to determine the MCID and DCID for each baseline level of nasal obstruction. However, in calculating the MCID of the different subgroups, we found that the MCID value for the subgroup with “moderate” nasal obstruction was less than the statistical error of measurement (SEM) of our instrument. We were therefore unable to present results for the MCID. However, the results of the DCID did not have this issue and are therefore still helpful for assessing individual results.

The DCID for each baseline level of nasal obstruction was determined using ROC curves and the user-developed Stata command cutpt, which empirically estimates the cut point for a diagnostic test. By default, the cutpt uses the Liu method (12), which maximizes the product of the test's sensitivity and specificity. The Youden and nearest methods were also evaluated, but since they yielded similar results, we opted to use the default Liu method. Both the sensitivity and specificity estimates for the DCID of each baseline level of obstruction are reported. Relative DCIDs were calculated by dividing the absolute DCID by the baseline level of nasal obstruction to determine the percentage improvement needed for each obstruction severity subgroup.

## Results

Nine hundred and thirty-four patients (663 men and 271 women) with a mean age of 37.7 years underwent endonasal septoplasty with or without turbinate surgery between 2014 and 2019 and completed both the preoperative and postoperative questionnaires.

The mean preoperative VAS score of the cohort was 75.1, with variations across baseline nasal obstruction subgroups: 55.5 for moderate, 78.7 for severe, and 94.0 for very severe (Table 1).

**Table 1:** Preoperative VAS scores, number of patients in each subgroup, and number of patients with status 1–5 (global rating).

VAS obstruction subgroup	Preoperative VAS, mean (SD)	*N* (%)	Outcome status, *n* (%)				
			1	2	3	4	5
Moderate (0–70)	55.5 (12.9)	332 (36%)	53 (16%)	169 (51%)	82 (25%)	18 (5%)	9 (3%)
Severe (71–85)	78.7 (4.2)	319 (34%)	44 (14%)	157 (49%)	93 (29%)	22 (7%)	3 (1%)
Very severe (86–100)	94.0 (4.8)	283 (30%)	43 (15%)	134 (47%)	66 (23%)	34 (12%)	9 (3%)
Total	75.1 (17.9)	934 (100%)	140 (15%)	460 (49%)	241 (26%)	74 (8%)	18 (2%)

Status 1: completely improved, status 2: much improved, status 3: somewhat improved, status 4: unchanged, and status 5: worse.

The numbers of patients in each obstruction severity subgroup with each outcome status (1–5) are also listed in Table 1.

Table 2 presents the preoperative, postoperative and change in VAS scores according to obstruction severity subgroup and outcome status (2–3). For those with outcome status 2 (much improved), the mean improvement in VAS scores varied from 32.7 to 67.2 and increased with increasing baseline obstruction severity. The postoperative scores for each obstruction severity group differed only slightly but increased from 22.8 to 26.2 with increasing baseline nasal obstruction. For outcome status 3 (somewhat improved), the mean improvement in VAS scores increased from 14.0 to 34.1 with increasing baseline obstruction severity, leading to a postoperative result varying from 40.8 to 60.0.

**Table 2:** Preoperative, postoperative, and change in VAS scores in each subgroup for statuses 2 and 3.

Status and VAS obstruction subgroup	*N*	Preop. VAS score	*N*	Postop. VAS score	*N*	Change in VAS score	*p*
Status 2 (much improved)							
Moderate (0–70)	169	55.5 (13.5)	168	22.8 (16.7)	168	32.7 (19.7)	<0.001
Severe (71–85)	157	78.7 (4.1)	156	24.6 (16.1)	156	54.1 (16.4)	<0.001
Very severe (86–100)	134	93.5 (4.9)	131	26.2 (17.7)	131	67.2 (18.3)	<0.001
Total	460	74.4 (24.4)	455	24.4 (16.8)	455	50.0 (23.1)	<0.001
Status 3 (somewhat improved)							
Moderate (0–70)	82	54.7 (13.1)	82	40.8 (17.1)	82	14.0 (18.3)	<0.001
Severe (71–85)	93	78.4 (4.7)	93	54.5 (17.5)	93	24.0 (17.6)	<0.001
Very severe (86–100)	66	94.1 (5.0)	66	60.0 (16.7)	66	34.1 (16.6)	<0.001
Total	241	74.7 (17.8)	241	51.3 (18.8)	241	23.3 (19.2)	<0.001

### Minimal clinically important difference

As described in the Statistical analysis section, the MCID value for the subgroup with “moderate” nasal obstruction was found to be less than the SEM of our instrument. We are therefore unable to present valid results for the MCID.

### Desirable clinically important difference

The VAS scores at the border between outcome statuses 2 and 3 were used to establish the DCID. This was done with the ROC, which gives the specificity and sensitivity of the values. In Table 3, we present the baseline and borderline (between statuses 2 and 3) scores together with the DCID (change in the VAS score from baseline) and the relative DCID (percentage change from baseline). The borderline VAS score increased from 28.0 to 38.0 with increasing baseline obstruction scores, resulting in the DCID scores ranging from 27.5 to 56.0 according to the severity of baseline obstruction.

**Table 3:** Preoperative (baseline) VAS score, borderline VAS score between statuses 2 and 3, specificity and sensitivity of the borderline score, DCID, and relative DCID of each subgroup.

VAS obstruction subgroup	Preoperative baseline VAS score	Borderline VAS score between statuses 2 and 3	Specificity/sensitivity	DCID change in the VAS score from baseline	Relative DCID % change from baseline
Moderate (0–70)	55.5	28.0	0.79/0.66	27.5	49.6
Severe (71–85)	78.7	34.2	0.90/0.76	44.5	56.8
Very severe (86–100)	94.0	38.0	0.94/0.78	56.0	61.3
Total	75.1	35.1	0.71/0.80	39.5	52.6

**Figure 1:**
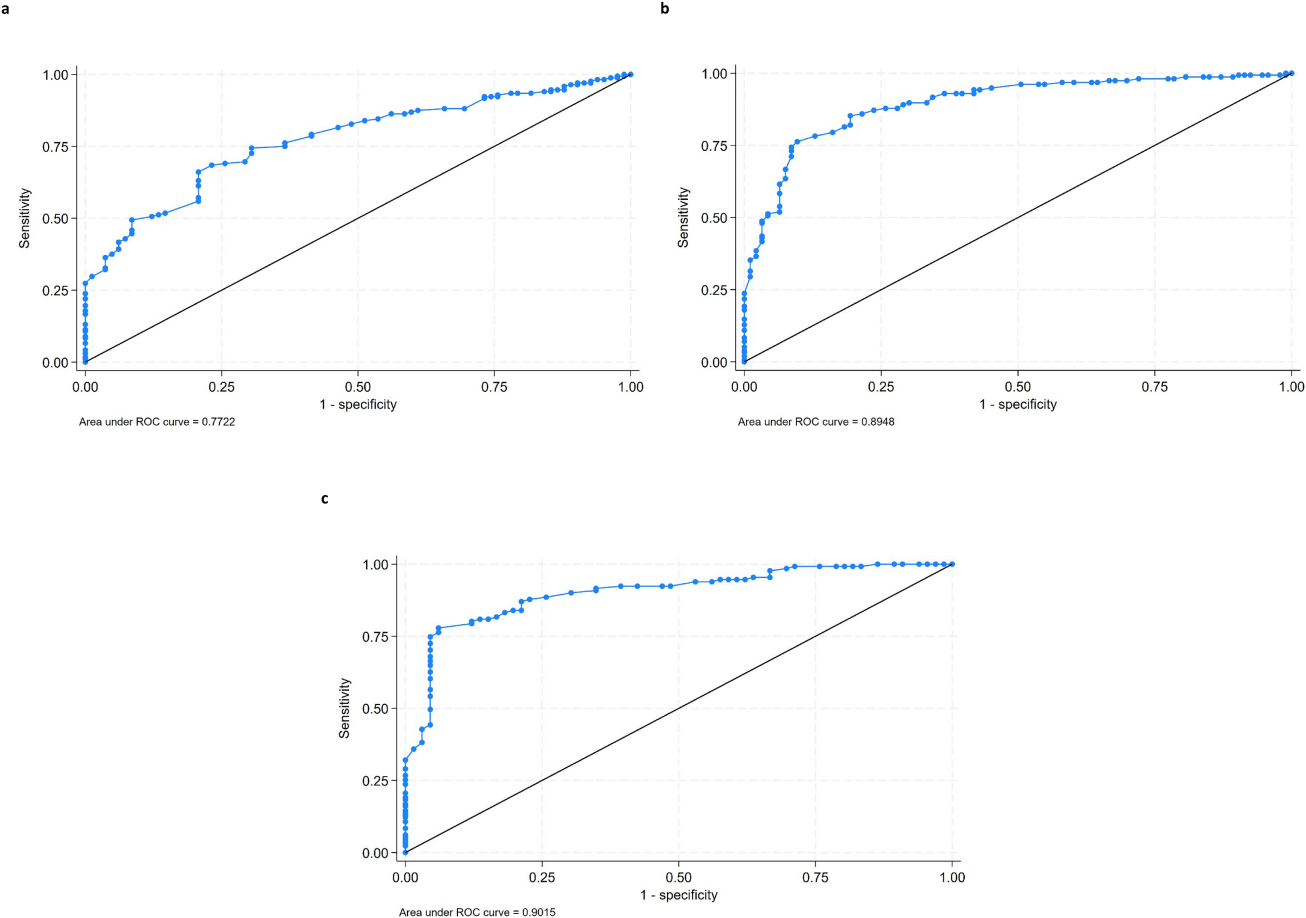
ROC curves of DCID for the VAS obstruction subgroups. Ratings are plotted according to sensitivity and specificity: (**a**) moderate (0–70), (**b**) severe (71–85), and (**c**) very severe (86–100).

ROC curves showing the results according to sensitivity and specificity for each VAS obstruction subgroup (moderate, severe, and very severe) are presented in Figure 1. The relative DCIDs varied from 49.6% to 61.3% with increasing severity of baseline obstruction.

## Discussion

The DCID varied across the three subgroups of baseline nasal obstruction severity. The DCID after septoplasty increased with increasing baseline ratings of nasal obstruction. The relative DCID also increased according to baseline obstruction severity, from 49.6% for moderate, 56.8% to severe, to 61.3% for very severe obstruction. Unfortunately, results could not be presented for the MCID due to the SEM of our instrument exceeding the MCID for one of the nasal obstruction subgroups. This measurement limitation highlights the importance of accuracy in symptom assessment. Nonetheless, as the MCID represents the “minimal” level of improvement considered clinically important, the larger DCID is likely a more useful standard for determining “surgical success” from the patient's perspective.

Several studies have evaluated septoplasty outcomes based on baseline ratings of obstruction. Hong et al. (13) in 2015 studied surgical outcomes after 3 months in 49 patients who underwent septoplasty and/or turbinoplasty using the nasal obstruction symptom evaluation (NOSE) scale. They concluded that “the baseline NOSE score was the sole factor that represented the subjective septoplasty outcome. Patients with more severe preoperative nasal obstruction may achieve a higher degree of satisfaction after septoplasty.”

Alakärppä et al. (14) in 2018 studied the result of septoplasty after 12 months in 76 patients using the Sinonasal Outcome Test-22 (SNOT-22). They found that the odds ratio of having a beneficial result was 10 when comparing patients with a baseline SNOT-22 score of 20 or more to those with lower scores. A baseline score of 30 was associated with the best postoperative improvement.

Pedersen et al. (15) in 2019 studied the results of septoplasty after 12 months in 888 patients. They subdivided the cohort into four categories according to the following baseline grading: none, mild, moderate, and severe obstruction. They defined postoperative improvement as a change in the grading of at least one step. The percentage of patients that experienced improvement increased according to the severity of the baseline rating: 31% among those with mild, 57% among those with moderate, and 81% among those with severe obstruction.

These three studies (13–15) are consistent with ours in finding that the higher the baseline ratings of nasal obstruction, the larger the improvement after septoplasty. As all of these studies used different instruments to rate obstruction severity, this finding seems independent of the instrument used. The main difference between these prior studies and ours was that the prior studies primarily used baseline obstruction severity as a predictor of septoplasty outcome and did not report the degree of improvement observed in each severity subgroup. In contrast, our study was primarily intended to serve as a guideline for assessing individual septoplasty results.

### Weaknesses

The primary weakness of this study was that it was based entirely on subjective data. Although patient-reported outcomes are important, we have no objective data to support our findings. An additional weakness is that the instrument we used to assess nasal symptoms had a degree of measurement error that prevented us from presenting valid MCID results for each level of nasal obstruction. Future studies using a different instrument may yield better results. The patients were asked to respond as on a normal day to avoid a temporary illness influencing the assessment. However, as this study aimed to compare VAS and global ratings, we did not account for the possible effects of smoking, allergy, use of medication, or quality of surgery on the ratings. The VAS scores were recorded prospectively, while the global outcome rating was done retrospectively. Retrospective ratings seem to correlate better to postoperative than to improvement scores. However, we previously found there to be a good correlation between the change in VAS scores and global ratings (5), so we believe that recall bias did not substantially influence the results.

## Conclusion

For assessing septoplasty results, it is important to find the level of improvement that the patient considers a surgical success. We have called this level the DCID. The DCID and the relative DCID increased with increasing baseline obstruction scores. Percentages of improvement as provided by the relative DCID can be used as guidelines to assess individual results of septoplasty. They can also be used in planning surgery for individual patients. Future studies are needed to determine the MCID for different levels of baseline nasal obstruction, as this study was unable to estimate this lower standard of clinically important symptom improvement.

## Data Availability

The original contributions presented in the study are included in the article, further inquiries can be directed to the corresponding author.
